# Cardiovascular and Autonomic Phenotypes Reveal Distinct Mechanisms of Sepsis Decompensation via Deep Learning

**DOI:** 10.21203/rs.3.rs-9136766/v1

**Published:** 2026-03-23

**Authors:** Tilendra Choudhary, Haoming Shi, Ayman Ali, Victor Moas, Omer T. Inan, Mihai V. Podgoreanu, Vijay Krishnamoorthy, Craig S. Jabaley, Craig M. Coopersmith, Michael R. Pinsky, Gilles Clermont, Rishikesan Kamaleswaran

**Affiliations:** 1 Department of Surgery, Duke University School of Medicine, Durham, 27710, NC, USA; 2 Department of Anesthesiology, Duke University School of Medicine, Durham, 27710, NC, USA; 3 Department of Biomedical Engineering, Duke University, Durham, 27705, NC, USA; 4 School of Electrical and Computer Engineering, Georgia Institute of Technology, Atlanta, 30332, GA, USA; 5 Department of Anesthesiology and Emory Critical Care Center, Emory University School of Medicine, Atlanta, 30322, GA, USA; 6 Department of Surgery and Emory Critical Care Center, Emory University School of Medicine, Atlanta, 30322, GA, USA; 7 Department of Critical Care Medicine, School of Medicine, University of Pittsburgh, Pittsburgh, 15213, PA, USA.

## Abstract

Sepsis heterogeneity reflects diverse etiologies and patient-specific physiological responses, motivating phenotype identification to enable precision therapeutics. However, most phenotyping approaches rely on intermittently sampled clinical variables, whereas continuously recorded physiological waveforms remain underutilized. We developed a deep-learning framework to derive physiological phenotypes from five-minute pre-onset electrocardiogram, photoplethysmogram and respiratory-impedance waveforms in 2,174 ICU patients meeting Sepsis-3 criteria. From these signals, 192 cardiorespiratory physiomarkers were extracted and embedded using a Feature Tokenizer Transformer encoder, which outperformed alternative representation methods. Consensus clustering identified four stable sepsis physio-phenotypes (SP-1–SP-4) associated with distinct autonomic and peripheral vascular signatures. Despite similar baseline severity and demographics, phenotypes differed significantly in mortality (19–29%), septic shock, vasopressor use and mechanical ventilation, with divergent 28-day survival trajectories (P<0.01). Explainable AI provided clinically interpretable characterizations, and a trained classifier enabled real-time bedside phenotyping. This framework establishes waveform-based phenotyping as a foundation for precision medicine in sepsis care.

## Introduction

Sepsis is a leading cause of morbidity and mortality in intensive care units (ICUs), affecting millions of patients worldwide annually^1,2^. Despite advances in early recognition and supportive care, sepsis management remains largely empirical, applying standardized protocols to highly heterogeneous patient populations^3^. This “one-size-fits-all” approach overlooks the substantial biological and clinical variability in patients with sepsis and may contribute to repeated failures of targeted therapies in clinical trials^4,5^.

Phenotyping—identifying subgroups of patients with shared characteristics—offers a promising path towards precision medicine in sepsis^6,7^. Recent efforts have leveraged electronic medical record (EMR) data, including laboratory values, vital signs, and clinical features, to derive sepsis phenotypes associated with distinct trajectories and treatment responses^8–10^. While these EMR-based approaches have yielded valuable insights, they rely on intermittently sampled, discrete variables that capture only snapshots of a patient’s physiological state. In contrast, continuous physiological waveforms, including electrocardiography (ECG), photoplethysmography (PPG), and respiratory signals (RESP), capture real-time cardiopulmonary dynamics and contain rich information about autonomic function, hemodynamics, and respiratory mechanics—features not reflected in conventional vital sign monitoring^11,12^. Despite their availability in modern ICU monitoring systems, these high-resolution data streams remain underutilized for clinical decision-making and phenotype discovery^13^.

Advances in artificial intelligence and deep learning now enable sophisticated analysis of complex, high-dimensional physiological data. Deep representation learning methods, such as variational autoencoders and transformer architectures, can learn compact, meaningful patterns from complex multimodal data while preserving critical nonlinear relationships^14,15^. Combined with unsupervised clustering, these techniques offer powerful tools for uncovering latent subgroups within heterogeneous patient populations.

Here, we introduce a novel framework for physiologic phenotyping (“physio-phenotypes”) of septic ICU patients using continuous multimodal waveforms and state-of-the-art deep learning methods. Our approach integrates: (1) comprehensive physiomarker extraction from multimodal continuous waveforms, (2) deep learning-based feature representation, (3) consensus clustering for robust data-driven phenotype identification, (4) Shapley Additive exPlanations (SHAP)-based explainable AI with large language model (LLM) interpretation for clinical characterization, and (5) validation across independent ICU cohorts. We hypothesized that this framework would reveal distinct sepsis phenotypes with distinct physiological profiles and clinical outcomes, potentially guiding interventions.

## Results

**Figure 1:**
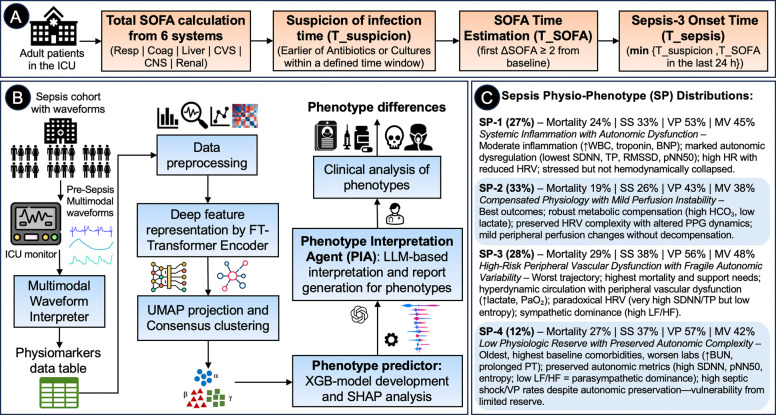
An overview of our phenotype derivation method and summary highlights of phenotyping results. (A) Flow diagram showing sepsis-3 time calculation; (B) Overall study pipeline of our phenotype derivation method showing physiomarker data extraction by multimodal waveform interpreter toolbox, data preprocessing, deep feature embedding generation, consensus clustering, LLM-based phenotype characterization, followed by phenotype analysis, and ML-based phenotype predictor development for future usage; and (C) Summary results of derived sepsis physiological phenotypes (SP), showing characterization and differences. Abbreviations: SS, septic shock status in 28 days; VP, vasopressor use in 28 days; MV, mechanical ventilation support in 28 days; HR, heart rate; HRV, heart rate variability; SDNN, standard deviation of NN intervals; TP, total power; RMSSD, root mean square of successive differences; pNN50, % NN intervals >50ms difference; LF/HF, low frequency/high frequency power ratio of HRV; PPG, photoplethysmography; BNP, B-type natriuretic peptide; WBC, white blood cell count; HCO_3_, bicarbonate; BUN, blood urea nitrogen; PT, prothrombin time; SOFA, Sequential Organ Failure Assessment; Resp, respiration; Coag, coagulation; CVS, cardiovascular system; CNS, central nervous system.

We described our dataset and study procedures in detail in Methods. A brief overview of our phenotype derivation method and a summary of phenotyping results are shown in Figure 1.

### Study Population and Cohort Characteristics

Among 4,806 ICU patients meeting Sepsis-3 criteria at the University of Pittsburgh (2016–2022), 2,174 sepsis encounters (2,106 unique patients) met all inclusion criteria and were retained for analysis. The most frequent exclusions were: absence of physiological waveforms during hospitalization (n=765), waveforms ending before sepsis onset (n=1,007), metadata/processing issues (n=577), and >80% feature missingness (n=89). The final cohort had a mean age of 60.2±16.2 years, with 58.4% male patients. The racial distribution was predominantly White (75.7%), with 12.2% Black patients. Overall in-hospital mortality rate was 24.1%. Baseline laboratory values immediately preceding sepsis-onset reflected typical early sepsis physiology, including elevated lactate (mean 2.2±1.5 mmol/L), mild acute kidney injury (creatinine 1.9±1.9 mg/dL), and moderate thrombocytopenia (platelets 222±118 × 10^3^/μL) (Table 1).

### Waveform Data Quality and Physiomarker Extraction

**Figure 2:**
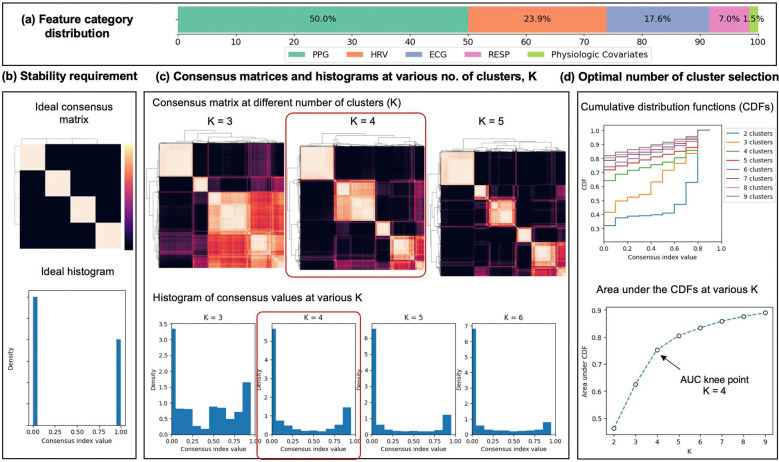
Stability analysis via consensus clustering and optimal number of clusters selection in our FT-T encoder-based phenotyping approach. (a) Initial feature category distribution; (b) The ideal consensus matrix (perfect block-diagonal structure) and its corresponding histogram, representing maximum stability where samples are consistently assigned to the same cluster across subsampling iterations; (c) Consensus value matrices and histograms obtained at various number of clusters, i.e., K, where they were highlighted at K=4 as the closest to the ideal; and (d) Selection of optimal number of clusters via area under the curves of cumulative distribution functions (CDFs) of consensus values.

High-resolution ECG, PPG, and RESP waveforms were successfully extracted for all encounters over the 5-minute pre-sepsis window. The Multimodal Waveform Interpreter generated 192 unique physiomarkers spanning: cardiac electrical activity, heart rate variability (HRV), PPG morphology and timing, and respiratory mechanics. After applying summary statistics (mean, 25^th^/75^th^ percentiles, skewness, entropy) and integrating baseline physiologic covariates, the initial feature space was obtained (distribution in Figure 2(a)). Following outlier removal, missingness filtering (>15% threshold), low-variance filtering, and correlation reduction (|r|>0.9), the final dataset contained 272 features with no missing values after imputation, listed in Supplemental Tables E1–E4.

### Four Stable Physio-Phenotypes Identified by Consensus Clustering

The training and validation results of the Feature Tokenizer Transformer (FT-Transformer/FT-T) Encoder are shown in Supplemental Figure E1. Consensus clustering with 80% resampling and 100 iterations on UMAP (Uniform Manifold Approximation and Projection)-projected FT-T embeddings revealed four stable sepsis physio-phenotypes (SP). Stability analysis of consensus value matrices, histograms, and cumulative distribution functions (CDFs) demonstrated: (i) clear block-diagonal consensus matrices structure and minimal ambiguous assignments at K=4 clusters (Figure 2(c)); (ii) plateauing of the CDF area under the curve beyond K=4, with a negligible increment for K≥5, (Figure 2(d)); (iii) favorable clustering metrics – Silhouette, Davies-Bouldin, and Calinski-Harabasz scores of 0.31, 1.079, and 1429.217, respectively, exhibiting further separability of the formed clusters.

**Figure 3:**
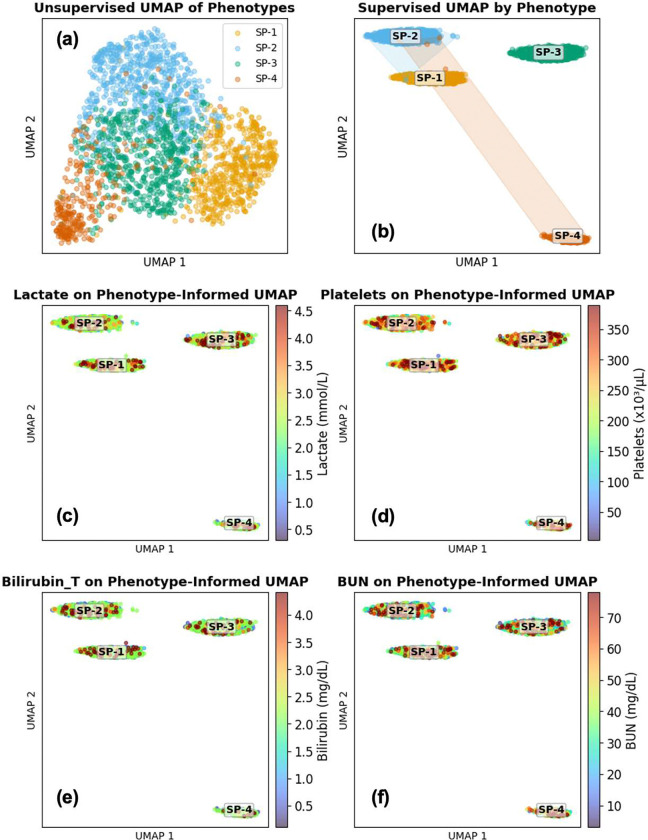
Visualization of identified sepsis phenotypes via UMAP embeddings. (a) Unsupervised UMAP of physiomarker features. (b) Weakly supervised UMAP colored by phenotype, with convex hulls and centroid labels SP-1–SP-4. (c–f) Post-hoc overlays of laboratory values on the supervised embedding from (b); labs were not used to construct the embedding. Color scales are clamped at the 95th percentile to highlight within-cluster variation and avoid distortion by extreme outliers, enhancing clarity.

**Figure 4:**
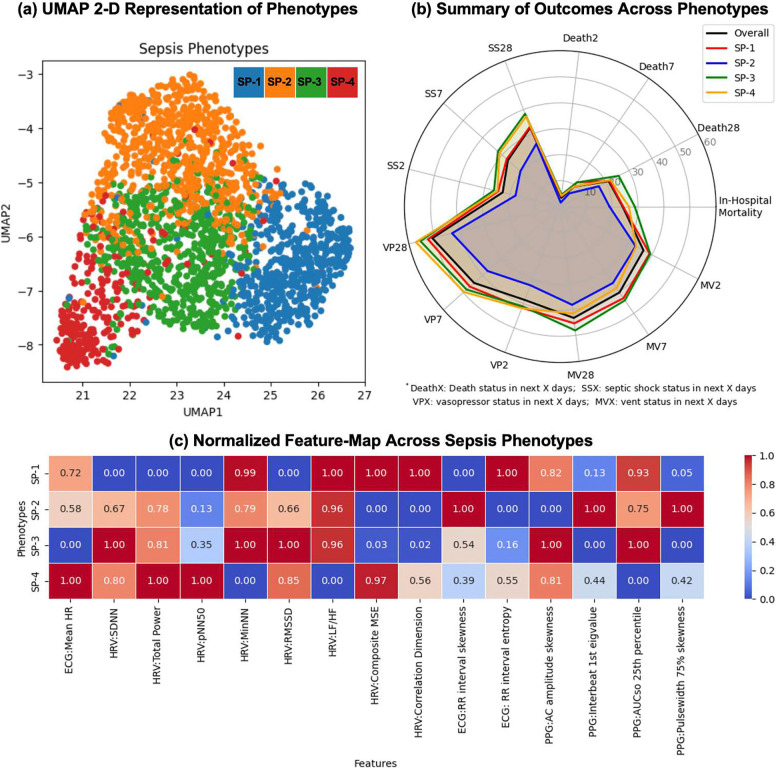
Multimodal physiologic phenotyping and outcome stratification in sepsis. (a) A 2-D UMAP projection of patients based on multimodal physiological features reveals four distinct sepsis phenotypes (SP-1, SP-2, SP-3, SP-4), demonstrating clear separation in latent feature space. (b) Radar plot summarizing short-term clinical outcomes across phenotypes, including in-hospital mortality, death at 2, 7, and 28 days (Death2, Death7, Death28), septic shock (SS2, SS7, SS28), vasopressor use (VP2, VP7, VP28), and invasive mechanical ventilation (MV2, MV7, MV28), with the overall cohort shown for reference (p<0.05). (c) Normalized feature map illustrating phenotype-specific differences in cardiac autonomic regulation (HRV magnitude and complexity metrics), heart rate (HR), ECG-derived temporal variability in cardiac cycles, and peripheral vascular dynamics from PPG (AC amplitude skewness and related indices). Collectively, these panels demonstrate that physiologic phenotypes correspond to distinct cardiovascular and peripheral vascular signatures that are associated with graded sepsis severity and clinical outcomes, with SP-2 exhibiting the lowest risk profile and SP-3 the most severe trajectory.

UMAP 2-dimensional projections showed visually distinct separation among the four phenotypes in the feature space (Figures 3(a) and 4(a)), with minimal overlap between clusters. The phenotypes were designated SP-1, SP-2, SP-3, and SP-4, with population prevalences of 26.7% (n=580), 33.2% (n=721), 27.6% (n=601), and 12.5% (n=272), respectively. Phenotype SP-4 represented the rarest but clinically distinct subgroup, while phenotype SP-2 was the most prevalent. Although SOFA scores prior to sepsis-3 onset were categorized into clinically meaningful severity groups (<2, 2–6, >6), their distribution did not differ significantly across clusters, suggesting that cluster assignment was not primarily driven by baseline organ dysfunction severity (Table 1). The unsupervised UMAP (Figure 3(a)) revealed natural clusters, while a weakly supervised UMAP (Figure 3(b); target weight=0.06) enhanced class separability, with convex hulls delineating phenotype territories and centroid labels providing spatial reference. Post-hoc overlays of laboratory biomarkers (Lactate, Platelets, Bilirubin, and Blood Urea Nitrogen (BUN), averaged over the first 24-h window after sepsis-3 onset) on the supervised UMAP embeddings—none of which were used in the clustering process—revealed phenotype-specific biochemical profiles (Figures 3(c)–3(f)).

Supplemental Figure E3 presents radar plots of demographic and mortality profiles for the four phenotypes, with the whole cohort (transparent dashed polygon) overlaid for comparison (see Table 1). Relative to the whole cohort, SP-2 was younger with the lowest mortality; SP-3 had the highest mortality; SP-4 was markedly older with more males and non-Hispanics; and SP-1 closely resembled the whole cohort but skewed slightly younger with fewer males.

Baseline comorbidity using the Charlson Comorbidity Index (CCI)^16^ was examined, which showed statistically significant differences across phenotypes. Supplemental Table E5 presents the CCI and age-adjusted CCI, along with the prevalence of various pre-existing conditions based on ICD-10 codes, with p-values. Phenotype SP-4 exhibited substantially elevated rates of key comorbidities relative to the overall population: congestive heart failure (46.1% vs. 26.3%), renal disease (42.1% vs. 29.0%), and diabetes (48.7% vs. 38.0%). The median age-adjusted CCI demonstrated a progressive increase across phenotypes: 4 (IQR, 2–6) in SP-1, 4 (IQR, 2–6.5) in SP-2, 5 (IQR, 2–7) in SP-3, and 7 (IQR, 5–9) in SP-4, indicating that SP-4 represents the phenotype with the greatest comorbidity burden.

### Physiological Signatures of Each Phenotype

The four identified physio-phenotypes demonstrated distinct physiologic signatures (Table 1, Figure 4(c)) based on SHAP analysis (Supplemental Figure E4; feature interpretations in Supplemental Tables E1–E4) and expert-validated LLM interpretation (Supplemental Table E7) by a Phenotype Interpretation Agent (PIA).

*Phenotype SP-1* (n=580, 26.7%) was characterized as reduced autonomic variability with cardiac irregularity. It was marked by profoundly reduced HRV across all domains. SHAP analysis identified the lowest HRV_SDNN (median 9.3 ms vs. 144.4 ms cohort-wide, p<0.001), minimal HRV_TP (0.026 vs. 0.086, p<0.001), near-absent pNN50 (0% vs. 9.8%, p<0.001), and markedly reduced RMSSD (8.9 ms vs. 199.5 ms, p<0.001). The highest entropy in RR interval (1.93 vs. 0.74, p<0.001) and correlation dimension (1.69 vs. 0.39, p<0.001) indicated irregular, chaotic cardiac rhythms despite reduced variability. SP-1 demonstrated severe autonomic dysfunction with loss of parasympathetic modulation and cardiac electrical instability^17,18^. Despite a younger age (55.8±16.0 years), mortality was near the cohort average (24.3%), raising the possibility that autonomic dysfunction may contribute to mortality risk independent of age.

*Phenotype SP-2* (n=721, 33.2%) was characterized as reduced complexity with altered cardiac rhythm. It exhibited elevated HRV magnitude with a high SDNN (median 184.6 ms vs. 144.4 ms cohort-wide, p<0.001). However, nonlinear complexity measures (Fuzzy Entropy and Katz Fractal Dimension (KFD)) were reduced, suggesting components of autonomic dysfunction, likely due to impaired autonomic regulation and increased physiological stress^19,20^. Notably, higher skewness in RR intervals (median 6.0 vs. 2.9, p<0.001) indicated irregular heart rhythms, which may reflect sepsis-induced cardiomyopathy or arrhythmias^21^, contributing to cardiovascular instability. Distinctively, this phenotype showed the lowest and most negative PPG AC amplitude skewness (−1.35 vs. −0.62, p<0.001) and highest PPG interbeat eigenvalue (4.58 vs. 2.71, p<0.001), indicating altered pulse morphology and increased beat-to-beat variability in peripheral vascular dynamics. These whole profiles suggested mild cardiovascular dysfunction, accompanied by mild autonomic dysfunction and altered peripheral perfusion, potentially reflecting peripheral vascular dysfunction or heterogeneous vascular tone. SP-2 had the lowest mortality (19.0%, p<0.001), suggesting a more favorable physiologic state despite sepsis.

*Phenotype SP-3* (n=601, 27.6%) was characterized as reduced complexity with altered pulse dynamics. The pattern of lower HRV complexity measures (HRV_HRV_CMSEn, HRV_HRV_CD, HRV_HRV_ShanEn) in SP-3 suggested impaired autonomic regulation and increased physiological stress. However, higher HRV_HRV_SDNN (median 271.7 ms vs. 144.4 ms, p<0.001) and RMSSD (373.8 ms vs. 199.5 ms, p<0.001) indicated a paradoxical increase in overall HRV, which might reflect compensatory autonomic responses or arrhythmia-related variability, demonstrating overall fragile autonomic variability. Additionally, SP-3 showed distinct PPG abnormalities (increased PPG_A_AC_skewness and decreased PPG_mean_slope_os_mean), suggesting changes in peripheral circulation, possibly due to vasodilation and early systolic upstroke caused due to altered arterial stiffness^22,23^, common in sepsis-induced hemodynamic instability^24^ – indicative of dysregulated peripheral vascular function. This phenotype had the highest mortality (28.8%, p<0.001), possibly due to its hyperdynamic cardiac state^25^. The dissociation between fragile autonomic regulation and peripheral vascular dysfunction might indicate advanced peripheral perfusion dysregulation or inappropriate vasodilation contributing to poor outcomes.

*Phenotype SP-4* (n=272, 12.5%) was distinguished by the oldest patient population (mean age 71.6±12.2 years, p<0.001) and remarkably elevated HRV magnitude and complexity such as higher pNN50 (71.5% vs. 9.8%, p<0.001), higher HRV_HRV_CMSEn (1.196 vs. 0.618, p<0.001), higher HRV_HRV_ShanEn, and lower HRV_HRV_LFHF (0.59 vs. 0.786, p<0.001), indicating dominant parasympathetic activity. However, the lower PPG_AUCso_nu_p25 (0.248 vs. 0.304, p<0.001) suggested altered peripheral perfusion and potentially increased arterial stiffness or reduced vascular compliance, possibly driven by systemic inflammation and altered peripheral arterial dynamics. SP-4 was therefore characterized by enhanced parasympathetic complexity with altered perfusion. In elderly patients, this pattern may reflect age-related autonomic remodeling or medication effects. Despite advanced age, mortality was intermediate (26.5%), indicating this autonomic pattern was not associated with excess mortality.

Normalized feature maps across phenotypes (Figure 4(c)) revealed complementary discriminative patterns across multiple dimensions. Supplemental Section E3 further explains the phenotypic differences with HRV magnitude and complexity. These multidimensional differences confirmed that the four phenotypes represented distinct physiological states rather than simply variations along a single severity axis. The physiological scope of PPG-derived features is also discussed in Supplemental Section E4.

### Clinical Outcomes by Phenotype

**Figure 5:**
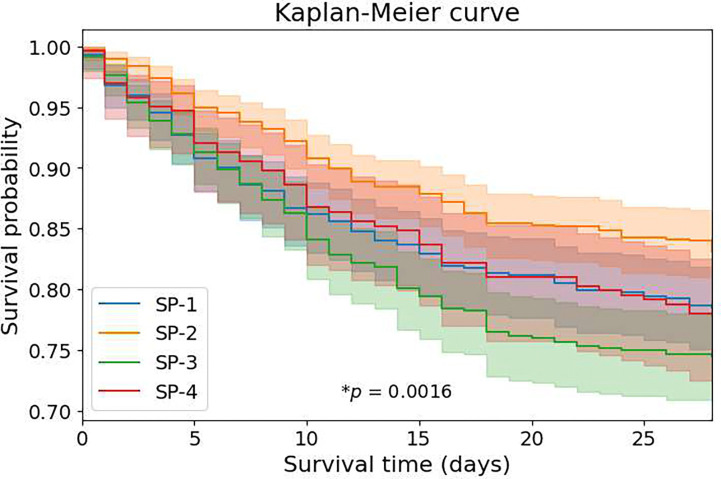
Kaplan–Meier survival analysis across sepsis phenotypes. Kaplan–Meier curves depicting 28-day survival stratified by physiologically derived sepsis phenotypes (SP-1, SP-2, SP-3, SP-4). Shaded regions represent 95% confidence intervals. Survival trajectories differ significantly across phenotypes (log-rank test, *p* = 0.0016), with phenotype SP-2 demonstrating the most favorable survival, phenotype SP-3 exhibiting the poorest survival, and SP-1 and SP-4 showing intermediate risk profiles. These results confirm that data-driven physiological phenotyping captures clinically meaningful heterogeneity in sepsis outcomes.

Clinical outcomes varied significantly across phenotypes (Table 1, Figures 4(b), 5). For in-hospital mortality, SP-2 exhibited the lowest (19.0%), phenotypes SP-1 and SP-4 showed intermediate (24.3% and 26.5%, respectively), while SP-3 had the highest mortality (28.8%). The 28-day short-term outcomes diverged markedly across phenotypes (Figure 4(b)). Mortality at 2, 7, and 28 days progressively increased for all phenotypes, with SP-3 consistently demonstrating the steepest mortality curve. By day 28, SP-3 reached approximately 26% mortality compared to 17% for SP-2. Kaplan-Meier survival analysis (Figure 5) confirmed significant survival differences (p<0.005), with SP-2 showing the best and SP-3 the worst survival within the first 10 days.

Septic shock prevalence at 2, 7, and 28 days (SS2, SS7, SS28) varied across phenotypes, with SP-3 and SP-4 showing higher proportions requiring early vasopressor support. Vasopressor use at corresponding time points (VP2, VP7, VP28) followed similar trends. In contrast, the need for invasive mechanical ventilation (MV2, MV7, MV28) was greatest in the SP-3 phenotype and second highest in the SP-1 phenotype. Overall, patients with the SP-3 phenotype required the most intensive cardiovascular and respiratory support throughout the observation period, whereas the SP-2 phenotype required the least. Support utilization was assessed as a binary variable (yes/no), without consideration of dose or intensity.

Phenotypes differed in severity trajectories despite similar baseline illness severity (e.g., creatinine, bilirubin, and lactate showed no significant differences before sepsis-onset, p>0.05), indicating that physiologic signatures captured pathophysiology not explained by conventional markers. However, laboratory markers showed statistically significant differences across phenotypes in a 24-hour window after sepsis-3 onset (Supplemental Table E6). BUN was highest in SP-4 (36.6±25.0 mg/dL vs. 31.8±25.3 cohort-wide, p=0.009), consistent with the older age. Troponin levels differed across phenotypes, with SP-1 showing the highest values (mean 131.42 ng/mL) and SP-2 the second highest (119.67 ng/mL), suggesting greater myocardial injury. Platelet counts were lowest in SP-2 and SP-4 (p=0.017), though all groups exhibited mild thrombocytopenia. In SP-3, lactate (2.631, p=0.016) and PaO_2_ (158.99, p<0.001) were highest, consistent with the most severe cohort.

### XGBoost Phenotype Predictor Enables Bedside Phenotyping

The eXtreme Gradient Boosting (XGBoost) classifier trained on physiomarkers (Supplemental Tables E1–E4) achieved an overall accuracy of 90.3% (80–20 train-test split) with balanced per-phenotype performance: SP-1 (precision 0.965, recall 0.940, F1 0.952), SP-2 (0.904, 0.891, 0.898), SP-3 (0.851, 0.904, 0.877), and SP-4 (0.891, 0.864, 0.877); full ROC, precision-recall curves (PRC), and confusion matrix are provided in Supplemental Figures E5–E6. SHAP analysis identified physiologically interpretable features as the primary discriminators for each phenotype (Supplemental Figure E4), confirming that the discovered phenotypes represent robust, learnable patterns in physiological data rather than clustering artifacts. The trained classifier enabled reliable phenotype assignment for real-time bedside deployment.

### FT-Transformer Outperforms Alternative Representations

The FT-Transformer encoder outperformed all alternative representation methods — Principal Component Analysis (PCA), Deep Variational Autoencoder (DVAE), and the no-transformation baseline — across various evaluation domains. Across clustering quality metrics, FT-T embeddings yielded the highest consensus stability, with a block-diagonal consensus matrix structure most closely approximating the ideal. Specifically, FT-T-derived clusters achieved a Silhouette score of 0.31, Davies-Bouldin index of 1.079, and Calinski-Harabasz index of 1,429.22, reflecting compact, well-separated clusters. Supplemental Figure E2 shows consensus matrices and histograms for all methods at K=4. FT-T generated the most stable clustering solution, with minimal ambiguity in sample assignments. Classifier-based validation further confirmed FT-T superiority: the XGBoost phenotype predictor trained on FT-T labels achieved the highest AUROC and AUPRC compared with classifiers trained on PCA, DVAE, or no-transformed features (Supplemental Figure E5), demonstrating that the FT-T latent space preserved physiologically meaningful substructure enabling accurate phenotype discrimination.

## Discussion

We developed the first comprehensive deep-learning framework for identifying physiologic-phenotypes of sepsis using continuous multimodal waveforms from the ICU. From 2,174 sepsis encounters, we discovered four distinct physio-phenotypes that capture differences in autonomic regulation, cardiovascular dynamics, and clinical outcomes (Figure 1(C)). Phenotype SP-1 (“Systemic Inflammation with Autonomic Dysfunction,” 26.7%) exhibited profoundly diminished HRV with disorganized cardiac rhythms, indicating severe autonomic failure and electrical instability. Phenotype SP-2 (“Mild Cardiac Instability with Altered Peripheral Perfusion,” 33.2%) demonstrated mild autonomic dysregulation with altered peripheral perfusion markers and had the most favorable outcomes. SP-3 (“High-Risk Peripheral Vascular Dysfunction with Fragile Autonomic Variability,” 27.6%) showed preserved central autonomic modulation but impaired peripheral vascular responsiveness (as evidenced by altered PPG-based dynamics), representing a paradoxical profile associated with the highest mortality (29%). SP-4 (“Low Physiologic Reserve with Preserved Autonomic Activity in Elderly Patients,” 12.5%) was characterized by dominant parasympathetic activity, high HRV complexity, and the highest comorbidity burden in the oldest patient-subgroup, with intermediate outcomes. Critically, these waveform-derived physiologic-phenotypes robustly stratified 28-day mortality (Kaplan-Meier analysis, log-rank p=0.0016), septic shock prevalence, and organ support requirements (vasopressors, mechanical ventilation) despite comparable baseline clinical severity. Specifically, lactate (p=0.064), creatinine (p=0.794), bilirubin (p=0.206), and SOFA scores (p=0.893) were similar across phenotypes (Table 1), demonstrating prognostic value independent of conventional severity assessments.

Our work advances sepsis phenotyping through four key innovations. *(a) Comprehensive Multimodal Waveform Interpretation:* Unlike prior phenotyping studies based on intermittently sampled EMR variables, our framework analyzes continuous ECG, PPG, and RESP waveforms to capture real-time autonomic, hemodynamic, and vascular physiology that is not observable through conventional vital signs or laboratory data, enabling granular cardiorespiratory phenotyping. *(b) Deep-Learning Representation of High-Dimensional Physiology:* Evaluation of four representation-learning approaches—including DVAE and FT-Transformer—showed that FT-Transformer generated the most stable and separable latent embeddings, outperforming alternative methods in consensus stability and classifier-based separability and revealing nonlinear physiological structure not captured by conventional dimensionality reduction. *(c) Robust Consensus Clustering:* Using 100 resampling iterations with agglomerative consensus clustering ensured that identified phenotypes reflected a stable data structure rather than model instability. Consensus matrices and CDF-based cluster selection reduced subjectivity and improved reproducibility. *(d) Explainable AI–Driven Interpretation:* Integration of SHAP-based explainable AI with domain-tuned LLM interpretation (Phenotype Interpretation Agent) translated complex model outputs into clinically interpretable physiological narratives. Physician-review further ensured clinical validity and relevance. Collectively, these innovations establish a scalable, interpretable framework for physio-phenotyping in critical illness.

Most prior sepsis phenotyping studies have relied on intermittently sampled clinical and laboratory variables from EMRs, identifying subgroups based on demographics, inflammatory markers, organ dysfunction, and metabolic features. Seymour et al. identified four EMR-based phenotypes in 20,189 septic patients using 29 clinical variables, demonstrating differential mortality and treatment responses^6^. Geri et al. identified five cardiovascular phenotypes in 360 septic shock patients on clinical and echocardiographic parameters^26^. While these studies identified clinically relevant subgroups, they cannot capture the high-resolution, real-time physiological dynamics embedded in continuous waveforms. Limited prior work has explored waveform-based phenotyping in critical illness: Moss et al. used heart rate characteristics to predict sepsis onset but did not perform phenotyping^27^, and Middleton et al. investigated PPG spectral analysis to assess peripheral vascular regulation in sepsis patients with no subgroup identification^28^. Unlike approaches based on discrete clinical measurements, our study is the first to integrate multimodal waveforms, extract comprehensive physiomarkers spanning cardiac, autonomic, and vascular domains, apply state-of-the-art deep learning representation with consensus clustering, and interpret phenotypes through explainable AI and clinical expertise. The 192 physiomarkers extracted from five-minute waveform segments provide granular information about autonomic regulation, cardiac electrical stability, and peripheral perfusion dynamics entirely invisible to conventional vital sign monitoring, establishing the clinical relevance of high-resolution waveform-based phenotyping in sepsis.

Across these phenotypes, SP-3 exhibited high-risk peripheral vascular dysregulation, demonstrating the highest mortality, septic shock burden, vasopressors, and ventilator use. Its physiomarker profile—elevated SDDN/TP, high PPG amplitudes, high LF/HF ratios, low HRV complexity (multiscale-entropy) with high HRV power—suggests a hyperdynamic yet dysregulated cardiovascular state with sympathetic overactivation and microvascular dysfunction, aligning with a non-cardiogenic shock etiology. Elevated lactate and PaO_2_ in the 24-hr post-sepsis window support the high proportion of septic shock and mechanical ventilation (Supplemental Table E6). Platelets were higher, while inflammatory markers were not disproportionately elevated, indicating that peripheral vascular dysfunction rather than systemic inflammation likely underpinned physiological deterioration. The high mortality in SP-3 may reflect metabolic failure seen in severe sepsis, where mitochondria shut down to preserve cellular integrity at the expense of organ function and microcirculatory flow^29^. Phenotype SP-2 exhibited the most favorable compensated physiology with higher bicarbonate, lower lactate, BUN and PT (Supplemental Table E6), indicating better acid-base balance and improved physiological status. Despite mild cardiac instability with altered peripheral perfusion inferred from waveform physiomarkers (higher SDNN, high RR-interval skewness, lower HRV complexity, and lowest skewness of PPG amplitude), this phenotype had the lowest mortality, vasopressor use, ventilator use, and septic shock prevalence, and represents a resilient physiologic subtype. SP-1 demonstrated autonomic failure with inflammatory stress. It showed profound autonomic dysfunction (lowest HRV SDNN, TP, and RMSSD) with irregular, entropy-rich RR dynamics, suggesting loss of parasympathetic modulation and electrical instability. Despite only modest laboratory abnormalities (elevated inflammatory and cardiac stress markers, including higher WBC counts and elevated troponin and BNP levels, intermediate lactate, relatively preserved BUN and creatinine), patients required substantial ventilatory support (second-highest) after sepsis onset, consistent with a hyperinflammatory state accompanied by early autonomic collapse. SP-4 consisted of predominantly older patients with the highest baseline comorbidity burden and preserved vagal tone. This phenotype showed high HRV complexity, elevated pNN50, SDNN, and lower LF/HF ratios, indicating dominant parasympathetic activity despite advanced age. However, peripheral perfusion indices were reduced, suggesting impaired cardiovascular resilience. Laboratory values in the 24-h window after sepsis-onset indicated reduced physiologic reserve: significantly higher BUN and PT indicated vulnerability to renal and coagulation stress, while lower platelet counts and modestly elevated lactate suggested limited metabolic buffering capacity. Despite preserved autonomic metrics, the underlying organ reserve appeared compromised. The dominant vagal tone pattern may reflect age-related autonomic remodeling, chronic rate-control or antihypertensive medications, or a distinct immunophysiologic response pattern in older adults, all deserving further investigation. Clinically, SP-4 exhibited the second-highest prevalence of septic shock and vasopressor use, consistent with diminished physiologic resilience — its substantial cardiovascular and renal comorbidity burden likely lowers the threshold for hemodynamic decompensation compared with younger, healthier patients. This contrasts with SP-1, where lower comorbidity burden implies greater baseline resilience and a higher systemic insult threshold for autonomous dysfunction. Importantly, our data distinguish HRV magnitude from HRV complexity as separate physiological constructs: elevated SDNN may reflect large but predictable oscillations from limited regulatory loops, whereas preserved nonlinear complexity reflects high-dimensional vagal-baroreflex-vascular interactions. Adaptive capacity likely requires both dimensions, forming a macro-physiologic signature of effective stress compensation (Supplemental Section E3). SP-2’s reduced nonlinear complexity, despite favorable outcomes and metabolic stability, represents compensated or outcome-based resilience sufficient for short-term stability but lacking multidimensional adaptability that characterizes intrinsic physiologic reserve. Conversely, SP-4’s preserved multiscale complexity reflects deeper mechanistic resilience despite advanced age and comorbidity. Together, these phenotypes reveal biologically coherent pathophysiologic signatures and suggest potential for phenotype-guided therapeutic strategies. Importantly, they likely reflect dynamic interactions among host reserve, infectious stressor characteristics, and illness stage, highlighting the value of tracking physiological trajectories rather than static subgroup assignments.

Identification of distinct physio-phenotypes with differential outcomes holds at least three significant implications for precision sepsis care. *First*, near-real-time phenotype assignment via the XGBoost-based predictor — operating on bedside waveform streams within minutes of sepsis recognition — enables continuous physiologic risk stratification (e.g., higher-risk SP-3 vs lower-risk SP-2) without additional laboratory input, potentially outperforming static scores such as SOFA or APACHE and improving triage, ICU resource allocation, and early warning systems. *Second*, phenotype-informed therapeutic strategies may explain heterogeneous treatment effects across prior sepsis trials. Autonomic and microcirculatory differences suggest differential responses to beta-blockade and autonomic modulation (SP-1, SP-3); microcirculatory and endothelial-stabilizing therapies with non-catecholamine vasopressors for SP-3; conservative vasopressor de-escalation for SP-2; and age-adjusted hemodynamic targets with judicious rate-controlling medications for SP-4. Such physiology-aligned treatment hypotheses may support precision-guided therapy. *Third*, physio-phenotypes may offer mechanistically grounded endpoints for clinical trials. Physiologic subtyping could reduce heterogeneity, reveal treatment-phenotype interactions, enable smaller proof-of-concept trials, and provide early surrogate markers of treatment response.

Several limitations warrant consideration. The retrospective design introduces potential bias and unmeasured confounding; waveform availability may limit generalizability; only a short pre-sepsis window was analyzed, limiting the assessment of earlier evolution or longitudinal phenotype dynamics; and additional waveform modalities (e.g., arterial pressure) were not incorporated. Applicability beyond Sepsis-3 definitions, to non-septic critical illness, or to diverse care settings remains uncertain. The high-dimensional feature space complicates clinical interpretability, despite SHAP-based prioritization. Treatment-phenotype interactions could not be assessed given limited intervention granularity; broader external multi-center validation is needed, and LLM-based phenotype interpretation requires further methodological evaluation. This work opens several promising avenues for future research. *First*, prospective validation with real-time phenotype assignment is needed to evaluate clinical impact on decision-making. *Second*, longitudinal phenotype characterization could identify trajectory-based subtypes and phenotype transitions during sepsis evolution and treatment. *Third*, integration with molecular, genomic, and metabolomic data could elucidate biological mechanisms and therapeutic targets. *Fourth*, investigation of phenotype-treatment interactions in randomized trial datasets could reveal differential intervention responses. *Fifth*, leveraging end-to-end deep learning, advanced interpretability (attention mechanisms or methods beyond SHAP), and federated multi-institutional training could enhance generalizability and clinical utility. *Sixth*, analyses using multiple or sliding windows, or earlier deterioration anchoring, could provide complementary insight into temporal evolution and phenotype stability. *Finally*, expansion to other acute critical illnesses could determine whether similar physiological subtyping approaches generalize beyond sepsis.

In summary, continuous physiological waveforms yield stable, mechanistically interpretable sepsis subtypes with distinct autonomic-cardiovascular signatures and differential clinical outcomes. These physio-phenotypes capture physiological dimensions inaccessible through EMR-based approaches, advancing the precision characterization of sepsis heterogeneity. The real-time XGBoost classifier renders phenotype assignment immediately actionable at the bedside. Prospective validation, longitudinal phenotyping, and integration with molecular data represent the next steps toward waveform-driven precision sepsis medicine.

## Methods

### Study Design and Setting

This retrospective observational study utilized an independently collected ICU cohort from multiple ICUs at the University of Pittsburgh (Pittsburgh, PA) between 2016 and 2022. Data were obtained under Institutional Review Board (IRB) approval (study number Pro00114885) with waivers of informed consent due to its retrospective design and minimal risk.

### Patient Population

We included adult ICU patients (≥18 years) who met Sepsis-3 criteria during their ICU stay^1^. Sepsis-3 was defined as documented or suspected infection, based on antibiotics and cultures administered within a specific time window, accompanied by an acute change in the Sequential Organ Failure Assessment (SOFA) score by ≥2 points^1^. The index sepsis-onset time was the earliest timestamp when these criteria were satisfied. Computation of Sepsis-3 time is shown in Figure 1(A).

Inclusion criteria required the availability of continuous physiological waveforms for the region of interest (ROI) – 5 minutes preceding Sepsis-3 onset. Exclusion criteria included: (1) absence of continuous physiological waveform recordings during hospitalization, (2) waveform recordings that ended prior to Sepsis-3 onset, (3) waveform recordings that began after Sepsis-3 onset, (4) missing or corrupted metadata/header files, (5) processing errors during feature extraction, and (6) >80% missingness across extracted features after quality filtering.

### Data Collection and Region of Interest

For each eligible patient, we extracted a 5-minute ROI immediately preceding Sepsis-3 onset. We acknowledge that sepsis onset is a clinical continuum rather than a discrete event, and that the Sepsis-3 timestamp represents the time when diagnostic criteria were first met in the EMR rather than the true biological onset of organ dysfunction. Nevertheless, a standardized temporal anchor was necessary to enable consistent waveform extraction across patients. The 5-minute pre-Sepsis-3 window was selected for several reasons: (i) it captures the physiological state immediately preceding documented organ dysfunction, minimizing confounding from post-recognition interventions (fluid resuscitation, vasopressors, antibiotics); (ii) it maximizes data availability, as many patients lack high-quality waveforms in earlier time periods; (iii) it provides a brief but stable snapshot of physiological patterns, balancing temporal resolution against signal variability; and (iv) it enables reproducible analysis across cohorts with different documentation practices. Although alternative windows may offer complementary insights, the pre-recognition window was prioritized to capture intrinsic physiological patterns during the transition to major organ dysfunction while ensuring data availability before clinical interventions that typically follow sepsis recognition. Continuous waveforms collected during this ROI included: single-lead ECG, PPG, and transthoracic impedance respiration (RESP) signals sampled at standard bedside monitor frequencies. Clinical data extracted from the EMR included: demographics, comorbidities, SOFA scores, physiologic covariates at sepsis onset, laboratory values, therapeutic interventions (e.g., vasopressors, mechanical ventilation), and clinical outcomes, such as in-hospital mortality, septic shock, and organ support within defined time windows. An overview of the study pipeline is illustrated in Figure 1(B).

### Waveform Processing and Physiomarker Extraction

We developed a comprehensive Multimodal Waveform Interpreter to convert raw waveforms into physiomarkers through six sequential stages:

- *Preprocessing:* Raw waveforms underwent quality control, including flatline and clipping detection, physiological outlier removal (z-score based), and resampling to uniform signal frequencies.
- *Denoising:* Median filtering removed high-frequency noise, followed by bandpass filtering with signal-specific cutoff frequencies (ECG: 0.5–40 Hz; PPG: 0.5–8 Hz; RESP: 0.1–0.5 Hz). Signal quality assessment quantified the proportion of high-quality data in each ROI using validated algorithms^30,31^.
- *Signal Derivation:* Generated derived signals, computed to expand physiological information content, included: ECG-derived respiration (EDR) using R-peak amplitude modulation, velocity plethysmogram (VPG, first derivative of PPG), and acceleration plethysmogram (APG, second derivative of PPG).
- *Delineation:* Fiducial points were detected in each waveform using validated peak detection methods^32,33^ – ECG: R-peaks, QRS complexes, and other wave boundaries; PPG: systolic/diastolic peaks, onsets, and dicrotic notches; RESP: inspiration/expiration transitions.
- *Feature Extraction:* From identified fiducial points, we extracted 192 unique physiomarkers spanning multiple physiological domains: heart rate variability metrics (time-domain, frequency-domain, nonlinear), PPG morphology and timing metrics (pulse width, systolic/diastolic timing and area, pulse statistics), respiratory dynamics (breathing rate, respiratory width, inspiration/expiration timing and ratio), and ECG-PPG coupling indices (pulse arrival time).
- *Summary Statistics:* To characterize the statistical distribution of each physiomarker over the 5-minute ROI, we computed 16 summary statistics for time-varying features: mean, median, standard deviation, mean absolute deviation (MAD), interquartile range (IQR), minimum, maximum, 25th percentile, 75th percentile, skewness, kurtosis, Shannon entropy, linear slope, lag-1 autocorrelation, Gini coefficient, and valid data length. Heart rate variability indices were excluded from summarization as they represent pre-aggregated measures. Five summary statistics were retained for analysis to minimize redundancy while preserving physiological diversity: mean (central tendency), 25th and 75th percentiles (dispersion and quartile spread), skewness (distribution shape/asymmetry), and Shannon entropy (signal complexity/uniformity). This selection ensured comprehensive data representation while minimizing redundancy.

### Data Preprocessing and Feature Selection

To complement waveform-derived physiomarkers, we incorporated baseline physiologic covariates—systolic blood pressure (SBP), respiratory rate (RR), and Glasgow Coma Scale (GCS)—as categorical indicators capturing core hemodynamic, respiratory, and neurologic status at sepsis onset. These covariates provide a discrete clinical context alongside continuous waveform dynamics. Each variable was encoded using clinically established thresholds: SBP ≤100 mmHg, RR ≥22 breaths/min, and GCS <15 (coded as 1 if abnormal, 0 otherwise), along with a composite score (0–3) representing the number of abnormal physiologic domains. Finally, after physiomarker extraction, a feature dataset was created with continuous values and categorical indicators, comprising 429 features across 2,174 sepsis patients. Preprocessing steps included:

#### Outlier Rejection:

Z-score standardization identified and excluded extreme outliers (|z| > 6) that likely represented artifacts or measurement errors.

#### Missingness Filtering:

Features with >15% missingness were removed to ensure sufficient data completeness.

#### Imputation:

Multiple Imputation by Chained Equations (MICE) with predictive mean matching^34^ addressed all population-level missingness.

#### Low Variance Filtering:

Features with near-zero variance (standard deviation < 0.01 after standardization) were excluded as they provided minimal discriminative information.

#### Correlation Reduction:

To minimize multicollinearity and feature redundancy, we computed pairwise Pearson correlations and iteratively removed features with absolute correlation >0.85, retaining the feature with higher mean absolute correlation to other variables.

#### Standardization:

Following feature selection, all variables underwent standard scaling (zero mean, unit variance) to ensure equal contribution regardless of measurement scale.

After preprocessing, the final dataset comprised 2,174 observations with 272 features, which served as input for subsequent data transformation and phenotype discovery. The details about the selected features are listed in Supplemental Tables E1-E4.

### Data Representation Learning

To identify optimal low-dimensional representations of high-dimensional physiomarker data, we used a Feature Tokenizer Transformer (FT-Transformer or FT-T) Encoder. The FT-T adapted transformer architecture for tabular data by treating numeric features as tokens^35^. Each of the 272 scalar features was linearly projected into a 128-dimensional token embedding space. A learnable class token (CLS) and learnable positional embeddings were added to the token sequence to encode feature identity. The token sequence passed through three stacked transformer encoder blocks, each comprising: Multi-head self-attention (4 heads, 128-dimensional model space with 32 dimensions per head), Feed-forward network (256 hidden units, ReLU activation), Residual connections and layer normalization, and Dropout regularization (rate=0.1).

Self-attention mechanisms enabled the model to learn adaptive feature interactions and hierarchical dependencies that conventional architectures might overlook. The output CLS token embedding, representing a global summary of all input features, was projected to a 32-dimensional latent embedding via a linear projection head.

Training proceeded in an autoencoder configuration, where a lightweight MLP decoder reconstructed the original 272 features from the 32-dimensional CLS embedding. Reconstruction loss (MSE) drove self-supervised learning of feature representations. Model training employed the Adam optimizer with an initial learning rate of 0.002 and adaptive reduction via ReduceLROnPlateau (factor=0.5, patience≈6), together with early stopping (patience=20) and batch size 32. The resulting 32-dimensional FT-Transformer embeddings captured complex inter-feature dependencies through learned self-attention over numeric feature tokens.

The performance of the FT-T encoder was evaluated and compared with the following three data transformation approaches: no transformation, Principal Component Analysis (PCA)^36^, and Deep Variational Autoencoder (DVAE)^37^. More details can be found in the Supplemental Section E1.

### Dimensionality Reduction and Consensus Clustering

The feature representation was performed using Uniform Manifold Approximation and Projection (UMAP) in 2 dimensions^38^. UMAP preserves both local and global structure in the data while enabling visual inspection of cluster patterns (n_neighbors=30, min_dist=0.1, metric=euclidean). To identify robust, stable phenotypes, we applied consensus clustering^39^ with the following specifications – Clustering Algorithm: Agglomerative hierarchical clustering with Ward linkage; Resampling Strategy: 100 iterations, each randomly sampling 80% of observations without replacement; Consensus Matrix: Computed as the proportion of resampling iterations in which each pair of observations was assigned to the same cluster; and Optimal Cluster Number: Determined by cumulative distribution function (CDF) analysis of consensus matrices, identifying the number of clusters where the CDF showed minimal increase with additional clusters, indicating stability. The consensus matrix diagonal structure and area under the CDF curve guided the selection of the final cluster number and the comparison of representation methods.

UMAP nonlinear dimensionality reduction was applied prior to consensus clustering, as direct clustering of the FT-T embeddings yielded unstable consensus matrices with poor block-diagonal structure. UMAP 2-D projection preserved the underlying data topology while enhancing cluster separability and stability, as demonstrated by clear consensus patterns. The 2D representation was selected based on its ability to yield minimal mean squared reconstruction error (MSE), consistent with higher dimensions (3–5D showed comparable MSE in preliminary analyses) as demonstrated in our prior phenotyping work^40^.

### Evaluation of Representation Methods

Clustering quality was compared across the four representation methods using: (a) consensus matrix stability measures (consensus matrix map and distribution); (b) phenotype predictability and cluster separability (areas under the ROC and PRC, obtained from a trained phenotype predictor); (c) Silhouette Score: Measures how similar observations are to their assigned cluster versus other clusters (higher is better)^41^; (d) Calinski-Harabasz (CH) Index: Ratio of between-cluster to within-cluster variance (higher indicates better-defined clusters)^42^; (e) Davies-Bouldin (DB) Index: Average similarity between each cluster and its most similar cluster (lower indicates better separation)^43^. After stability and reliability ((a) and (b)), the complementary metrics ((c)–(e)) further assessed cluster compactness and separation across representation methods.

### Phenotype Characterization and Interpretation

#### XGBoost Phenotype Predictor:

Following phenotype assignment, we trained an XGBoost multi-class classifier^44^ from the 429 preprocessed features to predict cluster membership. This model served multiple purposes: (1) validate phenotype reproducibility, (2) assess feature importance for phenotype discrimination, and (3) enable phenotype prediction in future external validation cohorts, making it suitable for clinical deployment. XGBoost was trained with default hyperparameters using an 80/20 randomized train–test split. Model performance was evaluated using precision, recall, F1-score, per-class accuracy, receiver operating characteristic (ROC), and precision-recall curves (PRC).

#### Explainable AI with SHAP Analysis:

For model interpretability and to understand which features distinguished each phenotype, we computed SHAP (SHapley Additive exPlanations) values^45^ for the trained XGBoost model. SHAP values, grounded in game-theoretic principles and computed using tree-based explainer, quantified each feature’s contribution to phenotype predictions, providing both global and phenotype-specific measures of feature importance. For each phenotype, we identified the top 20 most discriminative features based on mean absolute SHAP values and characterized their directional effects (positive or negative SHAP values indicating increased or decreased feature values driving phenotype assignment).

#### Phenotype Interpretation Agent (PIA):

We developed a Phenotype Interpretation Agent using domain-tuned large language models (LLMs) to generate clinically meaningful characterizations of identified phenotypes in an exploratory manner. Specifically, we used the GPT-4o (~200B, large model) from OpenAI as a zero-shot base LLM model in our study. For each phenotype, the PIA received (a) Top 10 discriminative features with SHAP importance scores, (b) Directional feature effects (increased/decreased relative to other phenotypes), (c) Summary statistics of clinical outcomes (mortality, interventions), and (d) Population prevalence. The LLM synthesized this information into coherent clinical narratives describing the physiological profile, potential underlying pathophysiology, and clinical implications of each phenotype. Critical care physicians independently reviewed the input prompt with the context and validated the LLM-generated phenotype characterizations, suggesting clinical accuracy and interpretability. The domain-specific, detailed prompts and feature contexts can be found in Supplemental Section E2.

### Statistical Analysis

Continuous physiomarker variables were presented as median[interquartile range] and compared using the Kruskal-Wallis test for non-normally distributed data. All categorical variables were presented as n (%) and compared using Chi-squared (χ^2^) tests. Clinical lab-results were presented as mean(standard deviation) and compared using One-way ANOVA tests. Clinical outcomes and therapeutic interventions, including in-hospital mortality, septic shock, vasopressor use and invasive mechanical ventilation, were compared across phenotypes. Survival analysis was performed using Kaplan-Meier curves with log-rank tests. Statistical significance was defined as two-tailed p<0.05. All analyses were performed using Python 3.10 with scikit-learn, TensorFlow, XGBoost, and statsmodels libraries.

## Supplementary Material

This is a list of supplementary files associated with this preprint. Click to download.

• SupplementalDocumentFinal.pdf

## Figures and Tables

**Table 1:** Summary of patient characteristics of the sepsis cohort and its phenotypes.

Parameters	Whole cohort	SP-1	SP-2	SP-3	SP-4	*p*-value
**count (%)**	2174(100)	580 (26.7)	721 (33.2)	601 (27.6)	272 (12.5)	-
**Mortality** *	507 (24.07%)	137 (24.29%)	132 (19.02%)	168 (28.77%)	70 (26.52%)	<0.001
**Age in years, mean(std)**	60.22 (16.24)	55.84 (16.03)	59.55 (16.02)	60.12 (15.95)	71.58 (12.22)	<0.001
**Males, count (%)**	1229 (58.36)	300 (53.19)	426 (61.38)	329 (56.34)	174 (65.91)	0.001
**Race: African American or Black, count (%)**	256 (12.156)	62 (10.993)	80 (11.527)	82 (14.041)	32 (12.121)	0.551
**Race: Caucasian or White, count (%)**	1595 (75.74)	431 (76.42)	527 (75.94)	430 (73.63)	207 (78.41)	
**Ethnicity: Hispanic, count (%)**	7 (0.33)	2 (0.36)	2 (0.29)	2 (0.34)	1 (0.38)	0.404
**Ethnicity: Non-Hispanic, count (%)**	1737 (82.48)	472 (83.69)	566 (81.56)	470 (80.48)	229 (86.74)	
**SOFA <2, count (%)**	1174 (54.00)	302 (52.07)	392 (54.37)	327 (54.41)	153 (56.25)	0.893
**SOFA 2–6, count (%)**	830 (38.18)	228 (39.31)	277 (38.42)	225 (37.44)	100 (36.77)	
**SOFA >6, count (%)**	170 (7.82)	50 (8.62)	52 (7.21)	49 (8.15)	19 (6.99)	
**Lactate, mean(std)**	2.183 (1.463)	2.208 (1.462)	2.112 (1.268)	2.297 (1.829)	2.063 (0.940)	0.064
**PaO2, mean(std)**	146.829 (78.699)	146.764 (76.223)	141.724 (77.129)	153.606 (83.730)	145.567 (75.890)	0.058
**Troponin, mean(std)**	1.834 (1.610)	1.937 (2.041)	1.746 (0.542)	1.762 (1.227)	2.003 (2.764)	0.035
**Bilirubin total, mean(std)**	1.703 (3.735)	1.825 (4.276)	1.852 (4.156)	1.539 (3.232)	1.409 (1.893)	0.206
**Creatinine, mean(std)**	1.863 (1.931)	1.884 (1.749)	1.811 (1.903)	1.913 (2.278)	1.842 (1.499)	0.794
**BUN, mean(std)**	32.134 (24.997)	32.073 (24.766)	30.316 (22.137)	32.099 (28.133)	37.125 (24.756)	0.002
**Platelets, mean(std)**	221.845 (117.619)	231.816 (126.889)	211.251 (104.473)	230.699 (125.225)	209.183 (109.443)	<0.001
**HRV_HRV_SDNN, m[IQR]**	144.351 [32.857,312.879]	9.280 [5.312,18.779]	184.614 [87.076,323.412]	271.662 [139.050,460.176]	219.034 [136.137,343.120]	<0.001
**HRV_HRV_TP, m[IQR]**	0.086 [0.039,0.131]	0.026 [0.009,0.056]	0.099 [0.065,0.138]	0.102 [0.068,0.140]	0.120 [0.084,0.152]	<0.001
**HRV_HRV_pNN50, m[IQR]**	9.795 [0.991,38.816]	0 [0,0.306]	9.028 [2.948,23.765]	25.362 [7.619,43.636]	71.455 [59.174,79.182]	<0.001
**HRV_HRV_MinNN, m[IQR]**	577 [484,684]	614 [534,712.250]	579 [494,662]	616 [522,719]	436.5 [381,503]	<0.001
**HRV_HRV_MaxNN, m[IQR]**	1704.5 [935,3110.750]	717.5 [596,852.500]	2357 [1502,3810]	2476 [1592,3666]	2019 [1288.750,3237.5]	<0.001
**HRV_HRV_RMSSD, m[IQR]**	199.492 [37.855,431.733]	8.879 [4.786,17.062]	249.684 [120.607,433.176]	373.849 [196.766,649.006]	320.049 [195.081,484.580]	<0.001
**HRV_HRV_LFHF, m[IQR]**	0.786 [0.489,1.257]	0.790 [0.403,1.469]	0.781 [0.562,1.315]	0.781 [0.477,1.192]	0.590 [0.402,0.848]	<0.001
**HRV_HRV_CMSEn, m[IQR]**	0.618 [0.245,1.116]	1.218 [0.719,1.352]	0.361 [0.165,0.618]	0.388 [0.162,0.618]	1.196 [0.937,1.386]	<0.001
**HRV_HRV_CD, m[IQR]**	0.387 [0.121,1.355]	1.687 [1.318,1.864]	0.160 [0.062,0.356]	0.193 [0.050,0.412]	1.022 [0.690,1.557]	<0.001
**ECG_HR_bpm_mean, m[IQR]**	86.204 [72.805,99.647]	87.925 [77.584,102.943]	86.066 [73.562,98.363]	78.513 [67.125,91.855]	91.517 [78.980,104.478]	<0.001
**ECG_T_rr_ms_skewness, m[IQR]**	2.894 [0.621,5.987]	0.107 [−0.210,0.518]	5.965 [3.631,9.943]	3.293 [2.275,5.818]	2.364 [1.421,3.670]	<0.001
**ECG_T_rr_ms_entropy, m[IQR]**	0.740 [0.307,1.456]	1.928 [1.454,2.059]	0.332 [0.114,0.659]	0.590 [0.251,0.890]	1.206 [0.880,1.552]	<0.001
**PPG_A_AC_mean, m[IQR]**	0.370 [0.325,0.405]	0.373 [0.340,0.414]	0.382 [0.355,0.407]	0.370 [0.270,0.399]	0.336 [0.294,0.370]	<0.001
**PPG_A_AC_skewness, m[IQR]**	−0.619 [−1.307,−0.042]	−0.384 [−0.978,0.047]	−1.345 [−1.990,−0.712]	−0.167 [−0.664,0.245]	−0.386 [−0.792,−0.102]	<0.001
**PPG_ppgInterbeat_eigval1, m[IQR]**	2.713 [0.950,5.076]	1.721 [0.425,4.710]	4.580 [3.015,6.221]	1.288 [0.496,2.921]	2.733 [1.120,4.301]	<0.001
**PPG_AUCos_nu_mean, m[IQR]**	0.149 [0.129,0.172]	0.149 [0.129,0.170]	0.139 [0.124,0.156]	0.159 [0.135,0.185]	0.163 [0.145,0.187]	<0.001
**PPG_AUCso_nu_mean, m[IQR]**	0.336 [0.315,0.364]	0.339 [0.318,0.368]	0.330 [0.308,0.349]	0.353 [0.330,0.380]	0.326 [0.302,0.347]	<0.001
**PPG_AUCso_nu_p25, m[IQR]**	0.304 [0.271,0.336]	0.315 [0.294,0.345]	0.302 [0.275,0.327]	0.320 [0.287,0.350]	0.248 [0.216,0.287]	<0.001
**PPG_PW_75_mean, m[IQR]**	187.629 [149.412,230.484]	186.727 [142.227,222.181]	180.212 [139.857,215.605]	206.682 [171.576,259.800]	187.296 [151.623,223.161]	<0.001
**PPG_PW_75_skewness, m[IQR]**	1.118 [0.165,2.759]	0.563 [−0.080,1.975]	2.540 [1.245,4.418]	0.448 [−0.107,1.250]	1.317 [0.506,2.784]	<0.001

For physiomarker variables, this table lists the medians and interquartile ranges (IQR: Q1-Q3) for each phenotype as well as for the whole cohort, whereas lab values are expressed in mean and standard deviation forms. The p-value is also provided for each variable to indicate the statistical significance of the differences among the phenotypes. For evaluating statistical significance, Kruskal-Wallis test was performed for continuous variables and Chi-squared test was used for categorical variables.

* Mortality was computed with respect to patients (not encounters). All the lab values and SOFA scores reported here, were collected immediately prior to sepsis-3 onset point.

**Abbreviations used** — count: total encounters, mean: average, std: standard deviation, m: median, IQR: interquartile range, BUN: blood urea nitrogen, ECG_HR_bpm: heart rate in bpm extracted from R-R (NN) intervals, HRV_SDNN: Standard deviation of NN intervals, HRV_pNN50: Percentage of NN intervals differing by >50 ms, HRV_MaxNN/HRV_MinNN: Maximum/minimum NN intervals, HRV_RMSSD: Root mean square of successive differences, HRV_LFHF: Ratio of low frequency (LF) to high frequency (HF) power, HRV_CMSEn: Composite Multiscale Entropy, HRV_CD: Correlation Dimension, HRV_TP: Total power, PPG_A_AC: Pulsatile or AC amplitude of PPG pulse, PPG_AUCso: Area under the PPG pulse between systolic peak to offset, PPG_interbeat_eigval1: First eigen value of covariance matrix created from PPG cycles, PPG_PW_75: pulse width at 75% of PPG level.

## Data Availability

Data and materials might be available upon request. The analysis code will be made available on GitHub upon acceptance.
